# Informed Consent in Patients with Aphasia: Scoping Review of Clinical Decision-Making Tools and Medico-Legal Issues

**DOI:** 10.3390/brainsci16060621

**Published:** 2026-06-10

**Authors:** Lara Brunasso, Rosario Maugeri, Giuseppe Pio Cipollina, Simona Pellerito, Stefania Zerbo, Ginevra Malta, Giovanni Grasso, Domenico Gerardo Iacopino, Antonina Argo, Giuseppe Davide Albano

**Affiliations:** 1 Neurosurgery Unit, University Hospital of Parma, 43126 Parma, Italy; brunassolara@gmail.com; 2 Neurosurgical Clinic, AOUP “Paolo Giaccone”, Post Graduate Residency Program in Neurologic Surgery, Department of Experimental Biomedicine and Clinical Neurosciences, School of Medicine, University of Palermo, 90133 Palermo, Italy; rosario.maugeri1977@gmail.com (R.M.); giuseppecipollina2@gmail.com (G.P.C.); giovanni.grasso@unipa.it (G.G.); gerardo.iacopino@unipa.it (D.G.I.); 3 Institute of Legal Medicine, Department of Health Promotion, Mother and Child Care, Internal Medicine and Medical Specialties, University of Palermo, 90133 Palermo, Italy; simona.pellerito00@gmail.com (S.P.); stefania.zerbo@unipa.it (S.Z.); ginevra.malta@unipa.it (G.M.); antonella.argo@unipa.it (A.A.)

**Keywords:** aphasia, informed consent, decision-making capacity, supported communication, speech–language pathology, medico-legal framework

## Abstract

**Highlights:**

**What are the main findings?**

- Available evidence suggests that aphasia may, in some cases, mask rather than eliminate decision-making capacity, potentially leading to inappropriate exclusion from informed consent processes.
- Supported communication strategies, including visual aids, simplified language, and structured assessment tools, can improve the evaluation of decision-making capacity in patients with aphasia.

**What are the implications of the main findings?**

- Multidisciplinary approaches involving speech–language pathologists should be integrated into informed consent and capacity assessment procedures.
- Standardized and validated aphasia-sensitive decision-making tools are needed to improve patient autonomy, clinical practice, and medico-legal protection.

**Abstract:**

Informed consent is a core ethical and legal requirement in clinical practice. For individuals with aphasia, language impairments can hinder communication during consent processes. However, aphasia is primarily a language disorder and does not inherently imply cognitive impairment, a distinction frequently overlooked in clinical and legal settings. This scoping review examines how decision-making capacity (DMC) is assessed and supported in adults with aphasia, and outlines the clinical, ethical, and medico-legal implications for consent procedures. The review followed PRISMA-ScR guidelines. A systematic search of biomedical and legal databases was conducted without time restrictions. Studies addressing informed consent or DMC in adults with aphasia were included and analyzed using a qualitative thematic approach. Out of 519 records, 9 studies (2010–2024) from Australia, Canada, the United Kingdom, and Ireland met inclusion criteria. These studies often referenced national legislation and rights-based frameworks to define clinical responsibilities. Three main themes emerged: (1) DMC assessments rely heavily on language, with limited involvement of speech–language pathologists (SLPs), despite their role in reducing bias; (2) supported communication strategies—such as simplified language, visual aids, alternative response formats, and structured tools—can uncover “hidden competence”; and (3) structural barriers, including time constraints, insufficient training, and limited access to aphasia services, restrict implementation. Current evidence remains limited, largely qualitative, and insufficient to support definitive clinical recommendations. Incorporating supported communication, multidisciplinary assessment, and thorough documentation may enhance fairness and legal robustness. Future research should focus on validating aphasia-sensitive tools and evaluating their impact on outcomes and medico-legal risk.

## 1. Introduction

Informed consent (IC) in clinical practice has evolved as a response to the long-standing issues caused between physicians and their patients by the traditional “paternalistic” model. Unlike its origins in clinical research, where it became a cornerstone of the Nuremberg Code [1], IC in clinical settings emerged largely from judicial precedents and the increase in medical malpractice ligation. Traditionally, patients seeking medical care entrusted physicians to act in their best interests, often accepting an imbalanced relationship in which consent was informal and did not meet modern standards [2].

According to Marten and Rich [1], the foundation of the IC doctrine in clinical care can be traced to three landmark legal cases—Mohr v Williams, Pratt v Davis, and Schloendorff v Society of New York Hospital—in which patients alleged that their physician had committed “battery” by performing procedures without explicit consent (which was considered non-consensual and offensive touching). These cases highlight the shift from implicit trust to a need for explicit authorization in medical decision-making.

Ethical principles of beneficence, justice and respect for autonomy underpin the legal and ethical framework of IC in clinical practice [3]. The protection of patient self-determination plays a central role, and it requires an assessment of the patient’s “decision-making capacity”, defined as the ability to make informed healthcare choices. According to Cocanour (2017) [3], there are three fundamental requirements for valid IC: the patient must be competent, adequately informed, and free from coercion. Effective communication should therefore be tailored to each patient’s cognitive and linguistic needs, ensuring a comprehensive understanding of all pertinent information [3].

As far as the Italian context is concerned, the IC was regulated—thanks to modern cultural, juridical, ethical, and social developments—with Law 219/2017, titled “Provision for informed consent and advance directives treatment”. This law focuses on a patient-centered standard of medical care, prioritizing the best interests of patients, in addition to regulating issues concerning end-of-life care [4].

Decision-making capacity has particular ethical and legal importance in certain clinical contexts—such as aphasia—where communication impairments may hinder the consent process and require nuanced and individualized evaluation.

Aphasia is an acquired language disorder resulting from damage to the brain’s language centers, typically in the dominant (usually left) hemisphere [5]. While stroke, especially involving the middle cerebral artery, is the most common cause, aphasia may also result from traumatic brain injury (TBI), brain tumors, infections, or progressive neurodegenerative disorders. The disorder can affect verbal or written expression, comprehension, or both, impacting multiple language functions and components such as semantics, grammar, phonology, morphology, and syntax. Importantly, communication impairment should not be automatically equated with impaired decision-making capacity. Decision-making capacity refers to the ability to understand relevant information, appreciate the consequences of a decision, reason about available options, and communicate a choice. In patients with aphasia, the primary difficulty often lies in the expression or comprehension of language rather than in the underlying cognitive processes required for decision-making. Consequently, patients may retain the capacity to make informed decisions even when they are unable to communicate effectively through conventional verbal interactions [6]. Failure to distinguish between linguistic deficits and cognitive incapacity may result in the inappropriate exclusion of individuals with aphasia from informed consent processes and healthcare decision-making. For this reason, supported communication strategies and adapted assessment approaches are essential to ensure that communication barriers do not mask preserved autonomy and competence [7,8].

The prevailing legal and ethical presumption is that individuals possess decision-making capacity unless proven otherwise [9]. Though often used interchangeably, capacity and competence are distinct: capacity is a clinical judgment made by healthcare professionals, whereas competence is a legal determination made by courts [10].

In a time where defensive medicine prevails—with the dawn of a new era in which the “doctor always knows best” era is coming to an end [11]—it is crucial to highlight the relationship between the quality of informed consent and the almost inevitable legal repercussions, which may lead to an increase in malpractice litigation.

To corroborate this, on a medico-legal level, negligence in properly informing a patient prior to any type of medical action can invalidate the informed consent, thereby exposing doctors to potential malpractice lawsuits [12].

A recurrent theme in empirical research is the causality between “communication gaffes” and insufficient informing, which subsequently affects patterns of initiating lawsuits over alleged negligence [13,14,15,16,17,18].

Although IC is a fundamental requirement in clinical care, five well-established exceptions exist: public health emergencies, medical emergencies, cases in which the patient waives consent, instances of “therapeutic privilege”, and cases involving patient incompetence [3].

This scoping review was conducted to comprehensively examine the status of patients with aphasia in contexts requiring IC. Specifically, it explores the ethical and legal dimensions of consent in aphasic individuals, methods of assessing decision-making capacity in patients with aphasia, perceived cognitive and communicative challenges, and relevant legislation.

A comparative analysis of European legal frameworks will also be provided, along with a discussion on evolving approaches that emphasize consensus-based strategies to better support and promote the autonomy of individuals with aphasia.

## 2. Materials and Methods

### 2.1. Study Design

This scoping review was conducted in accordance with the PRISMA-ScR (Preferred Reporting Items for Systematic Reviews and Meta-Analyses extension for Scoping Reviews) guidelines [19]. The completed PRISMA-ScR checklist is provided as Supplementary Material S1.

A formal review protocol was not registered because scoping reviews are generally not eligible for registration in PROSPERO. The aim of this study was to explore the legal and clinical literature addressing the capacity of individuals with aphasia to provide informed consent, with a focus on legal definitions, assessment methodologies, and comparative legislation.

### 2.2. Search Strategy

A comprehensive literature search was conducted in accordance with the PRISMA-ScR guidelines using the following electronic databases: PubMed, Scopus, Web of Science, and Google Scholar. Studies published in English or Italian were considered, with no restriction on publication date. The final search was performed on 30 October 2025.

The search strategy was developed by G.P.C. and S.P. and combined the following keywords and MeSH terms: [“aphasia”] AND [“informed consent” OR “decision-making capacity” OR “legal competence” OR “communication disorders” OR “healthcare law”]. In addition, the reference lists of relevant articles were manually screened to identify further eligible studies.

Study screening, eligibility assessment, and data extraction were conducted independently by multiple reviewers. Any disagreements were resolved through discussion and consensus among the authors. Given the exploratory nature of this scoping review, the search strategy was intentionally designed to broadly identify literature addressing clinical, ethical, and medico-legal aspects of aphasia and informed consent.

### 2.3. Eligibility Criteria

Studies were included if they:
  - Discussed aphasia in relation to informed consent or decision-making capacity;
  - Addressed legal, ethical, or clinical aspects of consent capacity assessment in patients with aphasia;
  - Presented clinical frameworks, communication-support strategies, or comparative legal analyses;
  - Were published in peer-reviewed journals.
Studies were excluded if they:
  - Were editorials, commentaries, conference abstracts, or narrative reviews without original data or relevant legal/clinical analysis;
  - Lacked full-text availability;
  - Involved exclusively pediatric populations;
  - Were published in languages other than English or Italian.

### 2.4. Selection of Sources of Evidence

**Figure 1:**
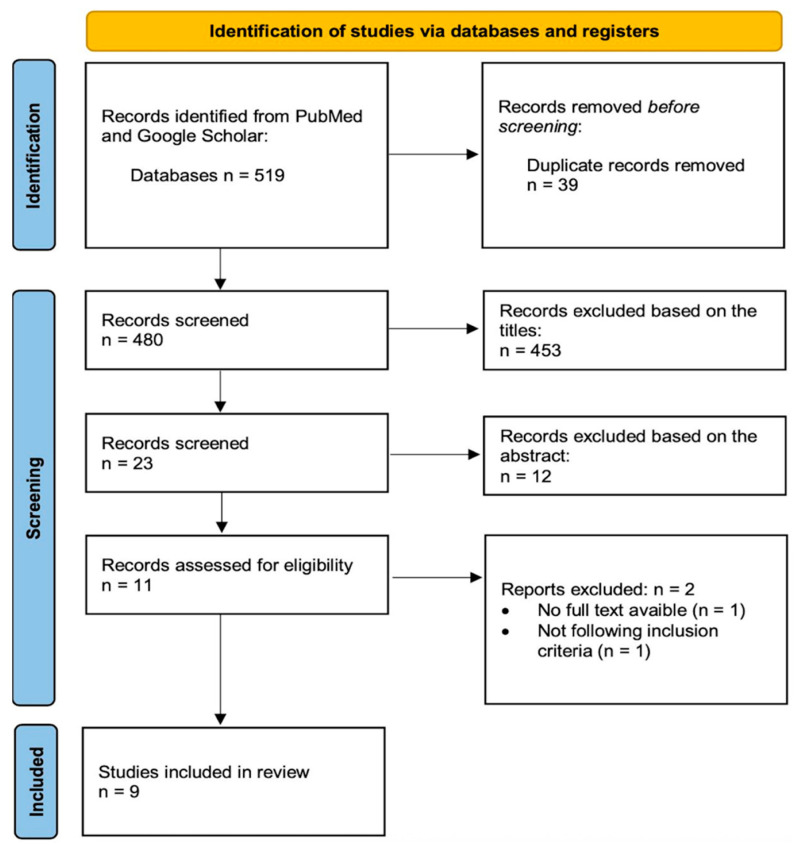
Research strategy diagram according to PRISMA guidelines.

After removal of duplicates, titles and abstracts were screened for relevance. Potentially eligible studies underwent full-text assessment according to the predefined inclusion and exclusion criteria. The study selection process is summarized in the PRISMA flow diagram (Figure 1).

### 2.5. Data Charting and Synthesis

Data were extracted and charted using a standardized approach. The following variables were collected from each included study:

- Country of origin;
- Study design;
- Objectives;
- Methodological characteristics;
- Legal or ethical frameworks referenced;
- Assessment tools or communication-support strategies adopted;
- Principal findings and recommendations.

A qualitative thematic analysis was conducted to identify recurring themes and gaps in the literature.

The findings were grouped into thematic categories reflecting the principal areas of interest:

- Definitions and classification of aphasia;
- Assessment of decision-making capacity;
- Supported communication tools;
- Legal and ethical frameworks;
- Comparative medico-legal considerations.

Consistent with scoping review methodology, no formal risk-of-bias assessment was performed, as the purpose of the review was to map the available literature rather than evaluate intervention effectiveness. However, given the clinical and medico-legal implications of the topic, we conducted a structured narrative appraisal of the included studies, focusing on study design, sample size, methodological limitations, directness of evidence, and relevance to informed consent and decision-making capacity in aphasia.

## 3. Results

The literature search yielded 519 records. After the removal of 39 duplicates, 480 titles were screened, and 453 were excluded for being unrelated to the review objectives. Abstract appraisal of the remaining 23 articles left 9 for full-text assessment (Figure 1).

Two full texts failed to meet ≥1 prespecified inclusion criteria, leaving 9 studies for inclusion in the scoping review (Table 1).

### Characteristics of the Included Studies

A total of 9 studies published between 2010 and 2024 were included in the review, covering research conducted in Australia (n = 2), Canada (n = 3), the United Kingdom (n = 3), and Ireland (n = 1).

Different methodological approaches were used: five were qualitative investigations utilizing semi-structured interviews, case-based commentaries, and thematic analysis; three had observational or quasi-experimental designs; and one was a single-case clinical report. Data sources varied across studies and included de-identified clinical transcripts, structured capacity assessments (e.g., CACE, MCA tools), linguistic and non-linguistic decision-making tasks, surveys, and multidisciplinary team observations.

All included studies explicitly engaged with the legal and ethical dimensions of decision-making capacity assessment in individuals with communication impairments, particularly aphasia or acquired brain injury (ABI). Legal frameworks referenced across the studies encompassed the Mental Capacity Act 2005 (UK), the Adults with Incapacity (Scotland) Act, the Alberta Adult Guardianship and Trusteeship Act (Canada), the Assisted Decision-Making (Capacity) Act 2015 (Ireland), and the United Nations Convention on the Rights of Persons with Disabilities (UN CRPD). In addition, five of the nine studies explicitly referenced clinical or professional practice guidelines, including those from the Royal College of Physicians, the Law Society of New South Wales, and various speech–language pathology (SLP) associations.

The included studies were not considered equivalent in evidentiary strength. Qualitative interview studies were interpreted as providing insight into professional perceptions and practice barriers, observational studies as documenting real-world service or assessment patterns, case-based reports as illustrative rather than generalizable evidence, and ethical or clinical commentaries as normative contributions rather than empirical proof. Accordingly, the synthesis was used to identify recurring concepts and gaps, not to establish effectiveness or generate definitive clinical recommendations.

The legal and ethical considerations were not merely contextual but actively informed the study designs and interpretative frameworks. Key themes included the operationalization of “functional capacity” in clinical contexts, the role of SLPs in interdisciplinary assessments, the risk of excluding individuals with language impairments from legal and healthcare decision-making processes, and the need for adapted communication strategies to ensure procedural fairness and autonomy. Collectively, these findings highlight the intersection between clinical assessment practices and legal obligations, emphasizing the necessity of legally informed, communication-accessible approaches in capacity evaluations.

Building on this, a closer examination of the included studies reveals three recurrent themes, each illustrated by complementary methodologies.

- Limited but pivotal SLP involvement. Two qualitative surveys of practicing speech–language pathologists (SLPs) (Suleman & Hopper 2015 [21]; Ferguson et al. 2010 [18]) show that therapists recognize how language-loaded capacity interviews disadvantage people with aphasia (PWAs), yet they are rarely formally asked to participate in these interviews. When involved, SLPs fulfill multiple roles—communication assessor, interpreter, educator, and patient advocate—but report inadequate training and unclear legal mandates.
- Supported communication reveals hidden competence. A single-case report (Maxwell et al. 2021 [26]) and two methodological papers (Kagan et al. 2020 [24]; Jayes et al. 2021 [23]) demonstrate that combining Supported Conversation for Adults with Aphasia (SCA™), the Communication Aid to Capacity Evaluation (CACE), and structured conversational analysis allows PWAs to express informed choices that standard cognitive screens routinely miss. Foulkes et al. 2024 [27] further validate Conversation Analysis as an objective tool for auditing real-world capacity interviews and shaping clinician training.
- Structural gaps in service delivery. Palmer et al. 2018 [22] documented that only 45% of 278 UK stroke survivors with aphasia had received any therapy in the preceding three months, at a median dose of 6.3 h—well below evidence-based targets. Morris et al. 2014 [20] identified seven legal-service needs for PWAs and highlight the difficulty of self-advocacy in acute care.

Taken together, available evidence suggests that aphasia may mask decision-making ability in some contexts. Although systematic use of supported-communication techniques and routine SLP input are essential to fair capacity assessments, both remain under-implemented.

## 4. Discussion

The included literature is clinically relevant but methodologically heterogeneous and remains limited in analytical depth. Ferguson et al. [18] used qualitative critical-incident interviews with speech–language pathologists and showed that SLPs may assume multiple roles in capacity-related situations, including assessor, consultant, educator, interpreter, mediator, and advocate. However, this study relied on retrospective professional accounts rather than direct observation of capacity assessments, and therefore provides insight into perceived clinical challenges rather than objective evidence of assessment validity.

Morris et al. [20] broadened the focus by examining legal and social justice needs in people with aphasia, identifying barriers related to health, finance, discrimination, abuse, and access to services. This study usefully situates aphasia within a broader access-to-justice framework, but it does not directly evaluate informed consent procedures or decision-making capacity assessments. Therefore, its relevance to clinical consent practice is indirect.

Suleman and Hopper [21] provide important qualitative data on SLPs’ perspectives, emphasizing that capacity assessments often rely heavily on language and may disadvantage people with aphasia. Nevertheless, the study is based on interviews with 15 SLPs in one Canadian jurisdiction, where legal recognition of SLPs as capacity assessors is limited. This raises questions about transferability to other healthcare and legal systems.

Palmer et al.’s [22] study differs from the other studies because it does not directly examine decision-making capacity or informed consent. Instead, it documents community speech–language therapy provision for stroke survivors with aphasia in the UK, showing limited therapy access and low treatment intensity. Its main contribution to the present review is therefore contextual: it highlights structural barriers that may also affect access to communication support during consent and capacity assessment.

Jayes et al. [23] represents one of the few studies evaluating a practical tool, namely a communication screening tool for mental capacity assessment. Its findings are particularly useful because they show that non-SLP healthcare professionals may fail to identify communication needs and may use screening tools inconsistently. However, the study also reported only moderate interrater reliability and poor criterion validity, confirming that available tools require further development and validation before they can support standardized clinical recommendations.

Kagan et al. [24] provide an ethically important discussion of “inherent competence” and communication support. However, it is mainly case-based and normative rather than empirical. While it strongly supports the involvement of SLPs and the use of supported communication, it does not provide comparative outcome data demonstrating that these approaches improve the accuracy or legal robustness of capacity determinations.

Maxwell et al. [26] illustrate how multidisciplinary functional assessment and supported communication may reveal decision-making capacity in an individual with Wernicke’s aphasia. This case is clinically valuable because it shows how language-loaded cognitive screens may misrepresent capacity. However, as a single-case report, it cannot establish generalizable effectiveness.

Finally, Foulkes et al. [27] provide direct observational evidence through conversation analysis of real-life capacity assessments. This study is important because it identifies how assessments are actually conducted in practice and shows variation in key phases such as option listing and information giving. However, it included only four recorded assessments, limiting generalizability.

Taken together, the literature suggests that communication barriers may interfere with the assessment of decision-making capacity in people with aphasia, but the strength of evidence remains limited. Across studies, there is consistency in identifying language-loaded assessments, insufficient professional training, limited SLP involvement, and the lack of standardized tools as recurring problems. At the same time, important inconsistencies remain regarding how aphasia is conceptualized, how cognitive impairment is accounted for, which professionals should lead assessment, and which communication strategies are most effective.

Although many individuals with aphasia retain full cognitive capacity, they are frequently excluded from the decision-making process due to their communication impairments. This exclusion is compounded by the absence of standardized assessment tools and the limited legal recognition of supported communication strategies.

Speech–language pathologists are instrumental in implementing communication approaches that facilitate valid consent. However, these methods have not been systematically incorporated into most legal or institutional protocols.

Aphasia primarily affects language functions, although some individuals may also present associated cognitive impairments depending on the underlying neurological condition. Indeed, aphasia is clinically heterogeneous, and in some individuals, language impairment may coexist with cognitive deficits that can genuinely affect decision-making capacity; therefore, assessments should remain individualized and multidisciplinary. Although it severely affects the ability to communicate, general cognitive functions—including intelligence, memory, and executive functions—often remain intact, as do decision-making capacities.

Nevertheless, a widespread but erroneous assumption that individuals with aphasia inherently lack the cognitive competence required for informed decision-making persists in clinical settings. This conflation of language deficits with impaired reasoning or judgment can result in the unjust exclusion of individuals with aphasia from critical processes such as IC and participation in their healthcare decisions [28].

### 4.1. Assessment of Decision-Making Capacity (DCM)

Beauchamp and Childress [29] defined seven prerequisites to obtain IC, the so called “elements of disclosure”: (a) competence in understanding and making decisions, (b) voluntary participation (in decision-making) and the elements of information, (c) disclosure (the material information), (d) recommendation (of a plan), (e) understanding (in disclosure and recommendation) and elements of consent, (f) the decision (in favor of the plan), and (g) authorization (of the chosen plan).

**Figure 2:**
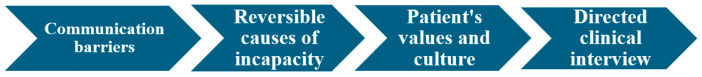
Stepwise approach to evaluating patient’s capacity, as proposed by Barstow et al. [10].

Regarding incapacity—possibly linked to various etiologies—a stepwise approach to evaluation is recommended (Figure 2).

Traditional evaluations of DCM, often based on verbal communication, can inadvertently penalize those with difficulties in language expression or comprehension. This poses a critical challenge: individuals with aphasia, while possessing intact reasoning and comprehension skills, may be misclassified as incompetent solely due to their inability to articulate or interpret complex language [30].

Without adaptation, traditional assessments risk violating fundamental ethical principles such as autonomy, justice, and beneficence. Emerging research and guidelines increasingly emphasize the importance of supported decision-making frameworks and the adoption of augmentative communication strategies and environmental adaptations, facilitated by professionals like speech–language therapists [31]. This approach represents a paradigm shift toward adapting the environment and communication supports to enable the person to participate meaningfully in decisions.

Traditional DMC assessments often rely heavily on verbal communication, potentially leading to misjudgment of a patient’s true capacity [21]. To address this, several tools have been developed to facilitate more accurate assessments of individuals with aphasia.

One such tool is the Communication Aid to Capacity Evaluation (CACE), which uses visual aids such as pictographs and simplified language to assess a patient’s understanding of key information such as the risks and benefits of medical treatments. Research has shown that when these visual aids are used, patients with aphasia demonstrate improved capacity to understand and make decisions about their care [26].

Another useful assessment tool is the Mental Capacity Assessment Support Toolkit (MCAST). The toolkit includes a structured interview process, along with visual and pictorial aids, to assess the patient’s understanding and ability to express a choice regarding treatment options. A key advantage of the MCAST is its ability to incorporate different forms of communication, such as gestures, pictures, and yes/no responses [25].

While tools like the CACE and MCAST represent significant advances, challenges remain. One of the primary issues is that many existing DMC assessment instruments were not originally designed with aphasia in mind. This gap suggests that further research and refinement are needed to develop more inclusive and tailored tools for assessing DMC in this population. It is crucial that new instruments not only assess the patient’s cognitive ability to make decisions but also consider their ability to comprehend and express their preferences in ways that align with their communication abilities [22].

In clinical practice, the involvement of a multidisciplinary team is essential for accurate and comprehensive DMC assessments in aphasic patients. Speech–language pathologists (SLPs), neurologists, psychologists, and other healthcare professionals must collaborate to determine whether a patient with aphasia has the cognitive capacity to make decisions. SLPs play a pivotal role in facilitating communication between the patient and the rest of the healthcare team. They employ techniques such as partner-assisted communication, augmentative and alternative communication (AAC) methods, and visual supports to help patients express their choices and preferences. For example, a trained communication partner can assist the patient in making their decision by interpreting non-verbal cues, gestures, or simple expressions [23].

Furthermore, the ability of people with aphasia to make decisions can be significantly enhanced when communication partners are trained and communication supports are provided that reduce linguistic and cognitive demands. Components of Supported Conversation Techniques for Adults with Aphasia (SCA™), such as augmenting information with different modalities, acknowledging competence, and allocating sufficient time, are identified as useful strategies. These strategies are often implemented by communication partners, most frequently SLPs and family members [28].

Given these considerations, a holistic approach to DMC assessment in aphasia is paramount.

Tailored, person-centered approaches, which incorporate visual aids, supported communication techniques, and multidisciplinary involvement, not only promote ethical care but also ensure that patients with aphasia retain their dignity and decision-making rights. Additionally, healthcare teams need to engage in ongoing training to recognize and accommodate the diverse communication needs of aphasic patients, ensuring that their decision-making capacity is accurately assessed and supported. Research and validated good clinical practices are urgently needed in these scenarios.

### 4.2. Legal and Ethical Considerations

Today, IC is a legal prerequisite for any healthcare intervention across European countries. While the ethical principles underlying IC—such as self-determination and the right to health—are universally recognized [32], national regulations differ (Table 2).

Moreover, a series of other functions are fundamental to the practice of IC, such as protection of both the doctor and the patient against malpractice litigations, prevention of medical abuse, self-ownership, the right to dispose of one’s own body and protection of personal integrity [29].

A comparative analysis of national laws highlights recurring themes essential to meeting the ethical and legal requirements of IC in clinical practice, chief among which are the ethical principles of beneficence, justice and respect for autonomy [3].

The legal frameworks reviewed share a common commitment to patient autonomy and informed consent but differ in how decision-making capacity is operationalized for individuals with communication disabilities. In common-law jurisdictions such as the United Kingdom and Ireland, capacity is generally assessed through a functional approach. The Mental Capacity Act 2005 and the Assisted Decision-Making (Capacity) Act 2015 emphasize decision-specific assessments and require reasonable support before a person can be considered incapable of making a decision. This approach is consistent with the communication-support strategies described in the clinical literature, including Supported Conversation for Adults with Aphasia (SCA), communication aids, and multidisciplinary assessment. By contrast, continental European frameworks, including those of Italy, Germany, Portugal, and the Netherlands, primarily focus on statutory protections of patient autonomy, informed consent, and access to healthcare information. For example, Italian Law 219/2017 and the German Patients’ Rights Act reinforce the patient’s right to receive information and participate in healthcare decisions. However, these frameworks provide limited guidance regarding the practical assessment of decision-making capacity in individuals with aphasia or other communication disorders.

A second area of comparison concerns professional liability. Both the Italian Gelli–Bianco Law (Law 24/2017) and the UK Montgomery v Lanarkshire Health Board decision highlight the importance of ensuring that patients receive information in a manner that allows for meaningful participation in decision-making. Although neither specifically addresses aphasia, both suggest that healthcare professionals have a responsibility to adapt communication when language impairments may compromise understanding and participation.

Despite these differences, a common limitation emerges across jurisdictions. While legal systems increasingly endorse patient autonomy, supported decision-making, and respect for persons with disabilities, none provides standardized aphasia-specific procedures for capacity assessment. As a result, clinicians are often required to translate broad legal principles into practice without validated communication-sensitive assessment frameworks.

Taken together, the reviewed literature suggests that the principal medico-legal challenge is not the recognition of autonomy itself, but the lack of standardized mechanisms to ensure that autonomy can be effectively exercised by individuals with aphasia. This gap between legal principles and clinical implementation remains an important area for future interdisciplinary research and policy development.

In fact, to protect the patient’s right to self-determination, it is necessary to define their “capacity”, implying the ability to make decisions. This, inevitably, leads to another recurrent theme, more specifically, the quality and quantity of the transmitted information. Once again, this is strictly linked to the type of relationship between the physician (source of the information disclosed and responsible for the entire process) and the patient. According to Cocanour (2017) [3], there are three fundamental criteria for valid IC: the patient must be competent, adequately informed and not coerced.

More specifically, three standards have been proposed for informing patients about a procedure: the professional practice standard; the reasonable person standard, which refers to what a typical reasonable person would be expected to know; and the subjective standard, which relates to what a specific patient would need to know. The latter is often considered the most challenging to apply in clinical practice.

Effective communication must therefore be tailored to the patient’s needs, ensuring that all material information relevant to the decision-making process is disclosed [3].

Finally, decision-making capacity, and its associated ethical and legal implications, becomes especially critical in certain clinical contexts—such as cases involving aphasia—where cognitive or linguistic impairments may complicate the IC process and necessitate careful, case-sensitive evaluation.

Generally speaking, the agreed-upon moral and legal presumption is that individuals should be considered capable of making decisions, unless proven otherwise [9].

It is important to then differentiate capacity from competence, although the terms tend to be used interchangeably. The former refers to a medical term determined by the treating physician, whereas the latter is a legal term determined by the court system [10].

Even though these statements should hold true in every situation, it is worth noting that there are five different exceptions that render informed consent unnecessary: a public health emergency, a medical emergency, cases involving patient waivers, “therapeutic privilege” circumstances and when the patient is incompetent [3].

In the specific case of patients with cognitive impairments, obtaining informed consent presents significant challenges, highlighting the ongoing struggle for healthcare providers to balance their patients’ rights to autonomy and self-determination with their duty to ensure appropriate medical treatment [38]. In particular, patients with aphasia—who are part of the broader language-impaired population—can be considered a “vulnerable population” [39]. This underlines, once again, the need to develop specific tools to ensure that such patients are properly informed and able to provide valid informed consent.

Given the absence of criteria and methodologies, researchers have proposed various ways to assess decision-making capacity [5].

As cited in [24], Zuscak et al. [40] propose a three-step decision framework that includes communication support strategies: Ref. [5] understanding relevant information, Ref. [41] appreciating consequences and reasoning and Ref. [42] communicating a choice.

These stages are similar to those typically described in both medical and legal contexts [24].

Focusing on the impact of aphasia on patients, a comparative analysis was conducted to identify strengths that may support the implementation of these approaches in both clinical practice and research.

In general, alternative consent must be sought when patients are deemed incompetent to consent and, consequently, unable to understand their condition, the treatment options and treatment consequences; furthermore, these situations call for the need for consideration of advance directives or legal proxies [38].

The approach to individuals with aphasia must be preceded by consideration of the “continuum of capacity”, which distinguishes three categories. At one end of the spectrum are individuals with very mild aphasia who are fully capable of making their own decisions; at the other are those with severe language impairments that compromise their ability to make informed decisions. In between are individuals who retain the capacity to make decisions but face difficulties expressing their choice due to their language impairments [43]. The last category, as cited in [31], is defined by Kagan [24] as people having “masked competence”.

Importantly, aphasia is clinically heterogeneous, and in some individuals, language impairment may coexist with cognitive deficits that can genuinely affect decision-making capacity; therefore, assessments should remain individualized and multidisciplinary. Some principles—such as modifications to both language and design—should be adopted to make written information more accessible to people with aphasia. Examples include limiting the amount of text on each page, simplifying the language, using larger fonts, and incorporating pictures to support the communication of key information [44].

The role of the family environment for patients with aphasia is crucial for both the care relationship and the process of providing information. Studies show that relatives or caregivers often form a “communication team” with the patient, helping to support their understanding and convey medical information without it, while not replacing the patient’s own will [45]. It is, however, essential that family involvement happen only with the explicit agreement of the patient, in order to respect their autonomy and self-determination. Otherwise, there is a risk that the patient’s voice may be overshadowed. From an ethical and legal point of view, the inclusion of family members in decision-making must be carefully documented in the medical record. This ensures transparency and also provides protection in case of medico-legal disputes. Brady and Kirschner point out that aphasia can be seen as a “family illness”, since it changes roles and decision-making dynamics within the household. Still, this does not mean that relatives can automatically replace the patient in making choices. Documenting the patient’s consent to involve a caregiver is therefore essential to balancing respect for individual autonomy with the practical need for communication support. In this way, the caregiver’s presence, when formally authorized, becomes both a valuable resource and a potential medico-legal concern.

The development and validation of tools to support clinical decision-making in patients with aphasia is an urgent priority. Shared decision-making is recognized as a cornerstone of person-centered care, yet the presence of communication disorders creates unique challenges for its implementation. Recent work has highlighted a variety of instruments designed to facilitate patient participation, but evidence of their actual impact on clinical outcomes remains scarce [46]. Without rigorous validation, it is difficult to determine whether these tools truly improve patient safety, therapeutic adherence, or satisfaction. Moreover, their medico-legal implications are particularly relevant: documenting the use of validated decision-making supports could offer clinicians greater protection in case of disputes, while the lack of standardized approaches risks increasing vulnerability. A systematic review by Durand and colleagues confirms that current evidence is insufficient to demonstrate a reduction in malpractice litigation through shared decision-making interventions [47]. This gap underlines the need for multicenter and longitudinal studies that can assess not only the clinical benefits but also the potential protective role of these instruments against malpractice claims. Future research should therefore aim to establish robust evidence linking validated decision-making tools with improved outcomes and clearer medico-legal safeguards, ultimately promoting both patient autonomy and professional accountability.

The ethical use of decision-making support tools in patients with aphasia requires careful consideration of clinical context. In emergency settings, clinicians act under the principle of necessity, making decisions that prioritize the patient’s best interest without delay. By contrast, in elective contexts the ethical imperative is different: healthcare professionals must adopt validated good practices that enable informed and autonomous choices, ensuring that patients with communication barriers are supported appropriately [39,48]. This distinction reflects core biomedical principles—respect for autonomy, beneficence, and justice—that require that patients be given every opportunity to participate in decisions that affect them [11]. Documentation is a critical safeguard: it not only demonstrates adherence to ethical and clinical standards but also functions as a protective measure in the event of malpractice claims. In fact, inadequate information exchange and poor documentation have been identified as major contributors to litigation, while shared decision-making and supportive tools may help prevent disputes [43]. The Italian Law 24/2017 (“Gelli–Bianco”) reinforces this dual perspective, requiring adherence to guidelines and good clinical practices while placing civil liability primarily on healthcare institutions rather than individual professionals. Validating these tools and integrating them into routine practice could therefore reduce litigation, promote cost savings, and, above all, enhance the quality and safety of care.

### 4.3. Limitations

This scoping review has some limitations. First, the available literature on informed consent and aphasia remains limited and methodologically heterogeneous, with most included studies consisting of qualitative investigations, case reports, or observational analyses. In addition, only studies published in English or Italian were included, potentially excluding relevant evidence from other linguistic and legal contexts. Moreover, because most included studies originated from English-speaking common law jurisdictions, the applicability of the discussed medico-legal considerations to continental European civil law systems remains limited and should be interpreted cautiously. Finally, consistent with the aims of scoping reviews, no formal critical appraisal of study quality was performed. The included studies are predominantly qualitative, observational, or case-based. No randomized or high-level interventional evidence currently exists in this field. The purpose of the review is to map the available literature rather than establish efficacy. The findings should therefore be interpreted as preliminary and hypothesis-generating. However, this scoping review underscores a disconnection between the clinical understanding of aphasia and legal and ethical frameworks governing IC. The current evidence base is characterized by small sample sizes, qualitative methodologies, case reports, and heterogeneous assessment approaches. Consequently, the findings of this review should be interpreted cautiously and primarily as exploratory evidence rather than proof of effectiveness.

## 5. Conclusions

This scoping review highlights the clinical, ethical, and medico-legal complexity of assessing decision-making capacity in individuals with aphasia. The available literature suggests that aphasia-sensitive communication strategies and multidisciplinary assessment may help reduce the risk of underestimating preserved decision-making abilities. However, current evidence remains limited, heterogeneous, and largely exploratory. Therefore, the findings should be interpreted as preliminary and hypothesis-generating rather than as a basis for definitive clinical standards. Further research is needed to validate standardized yet person-centered assessment tools, evaluate their impact on patient autonomy and care quality, and clarify their potential medico-legal implications.

## Figures and Tables

**Table 1:** Key findings of the included studies.

Authors (Year)	Country	Study Type	Objective	Methods/Data Source	Legal Considerations/Frameworks Referenced	Key Recommendations and Communication Strategies
Ferguson et al. (2010) [18]	Australia	Qualitative interview study	To identify key issues SLPs face regarding decision-making capacity in clients with aphasia and to describe their related practices qualitatively	Semi-structured interviews using the Critical Incident Technique were conducted, with verbatim notes taken during interviews and analyzed qualitatively using NVivo.	Enduring power of attorney provisions; Law Society of NSW (2003) guidelines for solicitors; UK Law Commission (1995) to support communication by using methods like AAC and simplified language or tasks	Recognition of SLPs’ active role; outline principles and scope of informal assessment; development of professional practice guidelines for SLPs. Multimodal communication strategies, including the use of pictures, photographs, writing, and natural gestures, to facilitate reliable yes/no responses.
Morris et al. (2014) [20]	Australia	Qualitative interview study	To provide preliminary information for the development of processes to ensure access to legal information and services for people with aphasia	Examined 167 de-identified transcriptions of recorded interviews with 50 participants with aphasia, 48 interviews with family members, and 69 interviews with SLPs. All participants spoke English; family and SLPs were nominated by the PwA. Aphasia severity (mild to severe) was assessed using the Western Aphasia Battery.	Disability Discrimination Act (1992); Ellison et al.’s (2004) framework, identifying seven legal need areas: accommodation, health, finance, discrimination, elder abuse, decision-making, and grandparenting	SLPs must be aware of the legal and access to justice challenges faced by PwAs and their families; SLPs must understand the legal and justice challenges PwAs face and act as key advocates, especially in acute care situations where PwAs struggle to self-advocate. Augmentative and Alternative Communication (AAC).
Suleman and Hopper (2015) [21]	Canada	Qualitative interview study	Explore SLPs’ perspectives on capacity assessments in patients with aphasia	Semi-structured interviews with 15 speech-language pathologists; interpretive-description analysis	Alberta Adult Guardianship and Trusteeship capacity definition; absence of SLPs in list of capacity assessors; Supported Conversation principles	Involve SLPs in capacity assessments; train assessors in aphasia-friendly communication; redesign materials and questions to reduce linguistic load. Supported Conversation for Adults with Aphasia (SCA), visual/pictographic aids, and material simplification using key words and gestures. These methods, alongside the Communication Aid to Capacity Evaluation (CACE), are used to reveal the patient’s true decision-making potential.
Palmer et al. (2018) [22]	UK	Observational analysis	Examine SLT provision to 278 community-dwelling stroke survivors with aphasia across 21 NHS trusts	Coded 278 community therapy records from 21 NHS trusts; descriptive statistics of goal types, dose, personnel	Royal College of Physicians stroke guideline; WHO-ICF framework; Malcomess Care Aims Model	Community provision is below evidence-based dose; adopt intensive and innovative delivery (groups, telehealth, computer therapy). Augmentative and Alternative Communication (AAC), total communication, and technology-supported strategies to improve participation and reduce the impact of language impairments.
Jayes et al. (2021) [23]	UK	Qualitative interview study	To provide an initial evaluation of the communication screening tool’s usability, interrater reliability and criterion validity	An electronic questionnaire and a documentary analysis; qualitative data were transcribed verbatim and analyzed thematically using a framework approach in QSR NVivo 9 software	MCA 2005; MCAST; SLT role; DCA 2007 and NICE 2018 guidelines	Enhanced Training and Support for HCPs; recognize the critical role SLTs play in mental capacity assessments; Investigating Effectiveness of Support Strategies; Advocacy for SLT Resources. Mental Capacity Assessment Support Toolkit (MCAST) incorporating alternative strategies such as reliable yes/no responses and augmentative methods like writing or pointing to photographs.
Kagan et al. (2020) [24]	Canada	Ethics/clinical commentary with cases	Discuss ethical implications of misjudging capacity and outline communication supports	Assessment of Supported Conversation training for healthcare providers	UN CRPD; Canadian clinical-ethics principles; concept of “reasonable accommodation” in capacity law	Emphasize “inherent competence”; provides stage-specific SCA-based strategies; emphasize moral imperative for SLP involvement in legal/clinical capacity work. Supported Conversation for Adults with Aphasia (SCA).
Kim et al. (2020) [25]	Canada	Ethics/clinical commentary with cases: “quasi-experimental design”	To examine PwA performance on linguistic and nonlinguistic decision-making tasks and explore its link to cognitive test results	16 PwAs and 16 age- and education-matched control participants completed three distinct decision-making tasks	MCA 2005	Language-heavy decision tasks may disadvantage PwAs; capacity assessments should include communication support like Augmentative and Alternative Communication (AAC).
Maxwell et al. (2021) [26]	Ireland	Single-case report	Demonstrate an MDT, function-based capacity assessment in a patient with Wernicke’s aphasia	Multidisciplinary functional assessment using the CACE, ward observations, and family meeting	Assisted Decision-Making Capacity Act 2015; UN CRPD; rejection of language-loaded cognitive screens	Use an MDT to scaffold communication; replace generic cognitive screens with functional tasks and CACE; ensure SCA and low-tech aids reveal true capacity. SCA, pictographic Communication Aid to Capacity Evaluation (CACE). Specific tools used included personalized wordbooks, written multiple-choice options, visual aids, and direct observation of activities of daily living (ADL) to reveal the patient’s true decision-making potential.
Foulkes et al. (2024) [27]	UK	Observational analysis	To objectively analyze real-life capacity assessments with ABI patients using CA in a hospital setting	CA was used to study interactions between healthcare professionals and patients during capacity assessments, guided by the MCA best practices.	MCA 2005; MCCP and NICE decision-making and mental capacity guidance	CA is a feasible empirical method for studying capacity assessments and can help develop evidence-based training for healthcare professionals (HCPs) in the assessments of decision-making capacity. Interactional and multimodal strategies, including conditional language, neutral option presentation (EQUIPOISE), and the use of gestures and patient view elicitors. These behaviors were combined with simplified terminology and “repeat back” understanding checks to facilitate capacity assessments for patients with acquired communication disorders.

**Table 2:** National differences in informed consent (IC) regulations across European countries.

France	**Law no. 303/2002—Act on the Rights of Patients and the Quality of the Health Care System** → Pertains to patient rights, healthcare quality and transparency and safety of care; involves a conciliatory approach for medical adverse events. **Law no. 370/2005—Act on the Rights of Patients and End-of-Life Decision-Making** → Deals with end-of-life decisions, shared decision-making for unconscious patients and duty of a palliative strategy; also addresses issues about IC, such as communication with patients, relatives and caregivers, and expression of will [32].
Spain	**Law 41/2002—Patient Autonomy and Health Documentation and Information, Related Rights and Obligations** → Regulates the rights and obligations of patients, users and professionals, considering the patient’s autonomy and the clinical information and documentation [33].
Germany	**Patients’ Rights** → Incorporated into the German Civil Code, aims to define patients’ rights while enhancing legal transparency. It codifies principles previously established through case law and formalizes the physician–patient relationship as a contractual obligation. Furthermore, it regulates informed consent and the scope and timing of patient information, and introduces duties of economic disclosure and error communication [34].
Italy	**Law 24/2017—On the Safety of Care, Patients’ Rights, and the Professional Liability of Healthcare Providers** → Also known as the “Gelli–Bianco Law”, it reformed the regulations governing liability for medical malpractice and patient safety. Its aim is to improve quality of care, patient safety and the medical liability system [35]. **Law 219/2017—On Informed Consent and Advance Healthcare Directives**→ Establishes a legal framework for informed consent and advance healthcare directives (ADs). It codifies the principles of patient autonomy, including the right to refuse or withdraw from any medical treatment, regardless of its potentially fatal consequences. It also guarantees that adults with legal capacity can predetermine their preference for future care. However, the law does not legalize euthanasia; rather, it emphasizes the provision of palliative care and deep sedation in end-of-life situations. Notably, the law is rooted in jurisprudential interpretation of constitutional principles, such as human dignity and personal freedom, while also aligning with international standards promoting respect for patient autonomy [36].
United Kingdom	The legal framework involves a balance between: **Common law principles** → Establishing fundamental concepts around an individual’s right to bodily integrity and decision-making in healthcare. Court decisions such as Collins v Wilcock serve to illustrate and develop these common law principles. **Legislation**: Acts of Parliament provide statutory rules. Key legislation includes the **Mental Capacity Act, 2005**, which defines, through statutory tests, the individual capacity to consent to treatment, and the **FamilyLaw Reform Act, 1969**, which addresses the capacity of minors aged 16 and over to consent to treatment [37]. **Montgomery v Lanarkshire Health Board** → This case drew fresh attention to informed consent and its scope [37], since the Royal Court recognized an individual’s right to self-determination in health-related decisions [32].

## Data Availability

No new data were created or analyzed in this study.

## Supplementary Materials

The following supporting information can be downloaded at: https://www.mdpi.com/article/10.3390/brainsci16060621/s1, Supplementary Material S1: Preferred Reporting Items for Systematic reviews and Meta-Analyses extension for Scoping Reviews (PRISMA-ScR) Checklist. Reference [49] is cited in the supplementary materials.

## Abbreviations

The following abbreviations are used in this manuscript:

AAC	Augmentative and Alternative Communication
CA	Conversation Analysis
CACE	Communication Aid to Capacity Evaluation
DMC	Decision-Making Capacity
IC	Informed Consent
MeSH	Medical Subject Headings
PRISMA-ScR	Preferred Reporting Items for Systematic Reviews and Meta-Analyses extension for Scoping Reviews
PWA	People With Aphasia
SCA™	Supported Conversation for Adults with Aphasia
SLPs	Speech–Language Pathologists
TBI	Traumatic Brain Injury
